# Territorial Hospitals and Nursing Service

**Published:** 1914-08-22

**Authors:** 


### TERRITORIAL HOSPITALS AND NURSING SERVICE.

In the list on p. 553 of last week's issue of The Hospital the name and address of the Principal Matron of the following general hospitals should be altered as below:

2nd London General Hospital of the City of London: Miss Darbyshire, St. Mary's Hospital, Paddington, W.

1st Western General Hospital, Liverpool: Miss Purves, 96 Princes Road, Liverpool.

1st Southern General Hospital, Birmingham: Miss Musson, General Hospital, Birmingham.

1st Eastern General Hospital, Cambridge: Miss C. C. Crookenden, Addenbrooke's Hospital, Cambridge.

2nd Eastern General Hospital, Brighton: Miss Bird, General Hospital, Croydon.

